# A Multi-Task Framework for Facial Attributes Classification through End-to-End Face Parsing and Deep Convolutional Neural Networks

**DOI:** 10.3390/s20020328

**Published:** 2020-01-07

**Authors:** Khalil Khan, Muhammad Attique, Rehan Ullah Khan, Ikram Syed, Tae-Sun Chung

**Affiliations:** 1 Department of Electrical Engineering, University of Azad Jammu and Kashmir, Muzaffarabad 13100, Pakistan; 2 Department of Software, Sejong University, Seoul 05006, Korea; 3 Department of Information Technology, College of Computer, Qassim University, Al-Mulida 51431, Saudi Arabia; Re.khan@qu.edu.sa; 4 Department of Computer Science, The Superior College, Lahore 54000, Pakistan; ikram.syed@superior.edu.pk; 5 Department of Computer Engineering, Ajou University, Ajou 16499, Korea; tschung@ajou.ac.kr; 6 Intelligent Analytics Group (IAG), College of Computer, Qassim University, Al-Mulida 51431, Saudi Arabia

**Keywords:** face image analysis, deep learning, face parsing, facial attributes classification

## Abstract

Human face image analysis is an active research area within computer vision. In this paper we propose a framework for face image analysis, addressing three challenging problems of race, age, and gender recognition through face parsing. We manually labeled face images for training an end-to-end face parsing model through Deep Convolutional Neural Networks. The deep learning-based segmentation model parses a face image into seven dense classes. We use the probabilistic classification method and created probability maps for each face class. The probability maps are used as feature descriptors. We trained another Convolutional Neural Network model by extracting features from probability maps of the corresponding class for each demographic task (race, age, and gender). We perform extensive experiments on state-of-the-art datasets and obtained much better results as compared to previous results.

## 1. Introduction

Face image analysis describes several face perception tasks, including face recognition, race classification, face detection, age classification, gender recognition, etc. These demographic attributes have been given immense attention in recent computer vision research due to large scale applications. Face analysis plays a crucial role in different real-world applications, including image augmentation, animations, biometrics, visual surveillance, human-computer interaction, and many other commercial applications. Despite significant research developments, face analysis is still challenging due to various reasons such as complex facial expressions, poor imagery conditions, and complex background. Face analysis has more complications in particular in the unconstrained and ‘in the wild’ conditions. Motivated by all the above reasons, we propose a multi-task framework that is targeting jointly three facial attributes, including race, age, and gender classification.

Besides a large number of benefits of an autonomous classification of gender, race, and age, there are certain social and ethical issues related to such classification. Some clinical practices believe that race and ethnic classification provides the crucial genetic surrogates that might be helpful in regimens treatment predictions. Due to such factors, and the increasing influence and attention given to the racial disparities in social and health, the definition of race have undergone scientific scrutiny [1]. Such practices have been seen from poor to modern societies. Moreover, the fields of cancer research, treatment, and prevention are facing the complexities of exploiting race and ethnic features for predicting outcomes medical decisions [2]. With reference to the gender classification, the authors in [3] report that female ratings of ethical judgment are consistently higher than that of males across two out of three moral issues examined (i.e., sales and retails) and ethics theories. The analysis of gender-based discrimination in [4] shows that worker characteristics and job search methods do account, although little of the gender gap in earnings.

There are multiple benefits to age, race and gender classification. With the increased use of smart devices, the autonomous recognition of age and gender can provide a large number of application-oriented benefits. One of the most beneficial is the recommendation systems. When the age and gender of a child are recognized, it should be helpful for many applications such as YouTube. YouTube can then use this information to recommend autonomously the age-based filtered videos. This can help in presenting related information to the user. Such recognition is also useful for autonomous parental controls of the websites and video services. The applications should thus provide a better experience, control, and security if the age of a particular user is correctly recognized. Similar other benefits can be exhibited by the computer-based applications if gender and age are recognized. Many shopping recommendation systems can present customized items to users just by recognizing their gender and age.

Compared to age and gender, we find that the justification and uses of race classification are expressively limited, and thus race classification is not only a sensitive and challenging matter, but many societies consider it an unethical process. Because it is believed that it could create and motivate social problems among masses. However, we believe that race classification can also be useful to several applications and scenarios. For example, the advanced countries experience an influx of illegal immigrants seeping into the country through several un-explored channels by the security agencies. The autonomous recognition and classification of the race at a number of locations inside the country can be very useful in this regard. Moreover, the border control can use such classification for better understanding and blocking of forged identities. Facebook and other social applications can use race classification for recommending related information, including but not limited to friends and products.

Typically, each of these facial attributes classification (race, age, and gender) are addressed individually through different set of methods [5,6,7,8,9,10,11,12]. We argue, all these tasks can be addressed in a single framework if sufficient information about different face parts is provided. In the proposed framework, we provide various face parts information through a prior segmentation model, which we develop through Deep Convolutional Networks (DCNNs). The psychology literature also confirmed the fact that different face parts help the human visual system to recognize face identity, and all face parts information is mutually related [13,14]. Therefore, the performance of all face related applications can be improved if a well-segmented face image having sufficient face parts information is given as input to the model.

The literature reports various methods to address human face analysis. Among all reported methods, face analysis through landmarks information is frequently used by researchers [8,15]. However, the performance in such cases is highly dependent on accurate facial landmarks information, which in real-world scenarios is again challenging [9,11,16]. These landmarks location identification is greatly effected with image rotation, occlusions, or if images are with poor quality. Similarly, landmarks extraction is again difficult if the images are collected in far-field imagery conditions. Due to all the problems mentioned above, we approach the face image analysis differently, i.e., providing prior face parts information through face image parsing.

We introduce a new framework in which face parts information is provided through a prior face segmentation model, which we develop through DCNNs. We address the three demographic tasks (race, age, and gender classification) through the face parts information provided previously. The proposed model is a joint estimation probability task that tackles it through DCNNs. The multi-task model can be formulated as;

(1) $$\left(r,a,g\right)=\underset{r,a,g}{arg max}p\left(r,a,g|I,B\right)$$

where race, age, and gender are represented by *r*, *a* and *g* respectively. The input face image is represented by *I* and the bounding box by *B* in Equation (1).

Multi-class face segmentation (MCFS) is already addressed by researchers [17,18,19]. Previously, face parsing was considered as three or sometimes four-class classification problem. In MCFS [17], face parsing was extended to six classes, including skin, hair, back, nose, mouth, and eyes. The MCFS [17] was developed through traditional machine learning methods (TMLMs). We addressed face parsing through DCNNs instead of TMLMs, obtained much better results as compared to previous results. Moreover, we extended our current research work to seven classes by adding eyebrow class. Additionally, MCFS [17] was evaluated on a minimal set of images, which we extended into three large datasets. We also extended our work to a joint task of race, age, and gender recognition. To summarize, the contributions of this paper are:

- We propose a new face parsing method through DCNNs, known as MCFP-DCNNs. We develop a unified human face analysis framework using the face parts information provided by a prior MCFP-DCNNs model. The multi-task framework is addressing the three demographic tasks (race, age, and gender) in a single architecture, which we named RAG-MCFP-DCNNs.
- We conduct detailed experiments on state-of-the-art (SOA) databases for face parsing, race, age, and gender classification. We obtained significant improvement in performance on both controlled and unconstrained databases for all four tasks.

The structure of the remaining paper is as follows: Section 2 describes related work for all the four cases, i.e., face parsing, race, age, and gender recognition. The databases used in the proposed work are discussed in Section 3. The proposed face parsing model is presented in Section 4. The multi-task face analysis framework is discussed in Section 5. All obtained results are discussed and compared with SOA in Section 6. The paper is summarized with some future directions in Section 7.

## 2. Related Work

Human face analysis is a well explored research area in computer vision. In this Section of the paper we review SOA methods used to address face parsing and remaining three demographic tasks.

### 2.1. Face Parsing

Face parsing methods can be categorized into two groups: local and global based methods. Local face parsing methods trained separate models for different face components such as eyes, nose, mouth, etc. For example, Luo et al. [20] proposed a method segmenting each face part separately. An interlinked DCNNs based method was proposed by Zhou et al. [21]. The approach proposed in [21] is benefiting from the complex sort of designing. The interlinked DCNNs can pass specific information between fine and coarse levels bidirectionally, consequently getting better performance at the expense of large computational cost and memory. A shallow DCNNs method having better computational cost as compared to the last mentioned method is proposed in [22]. SOA accuracy is obtained with [22] having a very fast running speed.

In global face parsing methods, a semantic label is predicted for each pixel over the whole image. Correlation between different face parts through different modeling methods is performed in some cases, as Epitome Model [23] and exemplar modeling method [24]. The underlying layout of the whole face image is performed through DCNNs. For example Aaron et al. [25] used facial landmarks information combined with DCNNs to address face parsing. Saito et al. [26] proposed that the computational cost of the face parsing can be much reduced with DCNNs, which makes a network fit for real-time applications.

Most of the methods mentioned above (algorithms with satisfactory performance) treated facial parts globally and inherently integrated them prior to the face image layout. Pixel labeling accuracy of all these methods was less because individual face parts were not focused upon. Moreover, most of these methods were evaluated with limited databases or images in the databases were collected in very constrained imaging conditions. Additionally, none of these methods addressed maximum face classes, but in most cases, only three or four classes were considered. We evaluated our framework on three large databases, namely, LFW-LP [27], HELEN [28], and FASSEG [29]. These databases include both low and high-resolution images. Images collected in very unconstrained conditions are also included. Moreover, unlike the previous methods considering a few semantic classes, we extend our face parsing work to seven semantic labels.

### 2.2. Race Classification

Race classification is a well-explored research area, but still, it is challenging due to certain reasons mentioned in the introduction portion of the paper. Recently, a method is proposed by Saliha et al. [30] for race classification. The proposed method combined local binary pattern information and logistic regression on a framework called Spark. Local binary patterns were used for feature extraction, and Spark’s regression for classification. The method was evaluated on two databases, namely FERET [31] and CAS-PEAL [32]. Two major races, Asian and Non-Asian, were included in the experimentation.

In holistic race classification methods, the face image is considered as one-dimensional feature vector, and some features are extracted. For example, Gutta et al. [33,34,35] used the RBF neural network and decision tree for race classification. The work was validated on FERET [31] dataset. Another race classification system was developed by Lu and Jain [36] through discriminant analysis. The system was tested on Asian and Non-Asian races. A support vector machine (SVM) classifier was used as a classification tool in another method proposed in [37]. The framework proposed in [37] was evaluated on a subset of face images from the FERET [31] database.

Manesh et al. [38] extracted face features from images through Gabor filter and used SVM for classification. The method proposed in [38] was evaluated on CAS-PEAL [32] and FERET [31]. Another approach [39] addressed the race classification through skin information and some secondary features such as lips and forehead information. For experiments, Yale [40] and FERET [31] databases were used. The framework classified five race classification, including Asian, American, Caucasian, African, and American. A comprehensive algorithm classifying three races Oriental, European, and African were classified by Salah et al. [41]. Face features were extracted through uniform local binary patterns combined with Haar Wavelet transform. For classification, K-nearest neighbors (KNN) was used. Some more methods addressing race classification through holistic methods can be explored in [42,43,44].

All the methods mentioned above are performed on a smaller or subset of a larger database. One method which was evaluated on comparatively larger dataset is reported by Xi et al. [45]. In this method, face information was extracted through color features. The performance of the framework was evaluated on the MBGC, having three classes of images. Han et al. [46] proposed another approach which was using biologically inspired features and hierarchical classifiers. Two large scale databases were used by the authors for experimentation, including MORPH2 and PCSO.

DCNNs are extensively used in different computer vision applications due to their excellent performance. A method proposed by Zhang et al. [47] used stacked spare auto encoding for features extraction. The classification was performed with regression Soft-Max method. Another flexible DCNNs method was proposed by Wei et al. [48]. Due to several different object segment hypotheses, this method was also called Hypothesis-CNNs-Pooling. The method proposed by Anwar and Nadeem [42] used DCNNs for feature extraction but performed classification through SVM.

### 2.3. Age Classification

Age classification can be studied both as regression and classification problem. Age is associated with a certain group in age classification, while the exact age of a person is estimated in the regression case. Our current research study regarding age is limited to classification only. Two survey papers are reported in literature [49,50], which addressed both age classification and estimation. The survey papers reported all the databases to date and also presented an overview of various age estimation methods as well.

Kwon et al. [51] proposed an age classification algorithm by extracting face information and training a classification tool. Face wrinkles information was used as features by the authors. Extension of the age classification using wrinkles information was done in another paper [52]. First facial features localization was performed, and then proper modeling strategy was adapted. Craniofacial growth information was extracted through anthropometric and psychophysical evidences, and modeling was performed. Accurate face features localization is necessary for the last mentioned approach. In some examples, when face features were not localized, the performance of the framework was drastically effected.

A new class of gender recognition methods was proposed known as AGing PatErn subspace methods [53,54]. Regression models were trained in these methods. For training regression models, features from face images that are related to aging were extracted. Both of these methods reported some excellent results as compared to SOA. Two main weaknesses faced by these methods, firstly, it was mandatory for face images to be frontal and well-aligned. Secondly, these methods are well suited for images collected in very controlled environmental conditions. The performance of these methods decreased as exposed to an unconstrained outdoor environment.

Another algorithm that used cost-sensitive hyperplanes information ranking way was introduced by Chang et al. [55]. It was a multi-stage learning algorithm which they named ‘a grouping estimation fusion’ (DEF). Another method that used features selection procedure was proposed in [56]. All the above-mentioned methods have shown good results in images collected in indoor conditions; however, when exposed to the real-world scenario, a drastic drop in performance was noted.

### 2.4. Gender Classification

Gender recognition received immense attention for many years due to its large scale applications in face analysis, particularly face recognition [57], soft-biometrics [58], and human-computer interaction [59].

Makinen and Raisamo [60] investigated gender recognition thoroughly in their work. Neural networks was used by early researcher to address gender classification [61]. However, very few (only 90) face images were used by Golom et al. [61]. Jia et al. [62] trained a gender classifier using four million weakly marked images. Similarly, Moghaddam and Yang [63] used SVM with some dimensionality reduction features for gender classification. Another paper [64] used Adaboost classifier for gender classification.

Antipov et al. [65] used deep learning architecture for gender recognition. The authors claimed that much improved performance can be achieved with less training data. The model was validated with CASIA [66] dataset, having 494,414 face images. Jia et al. [62], in another paper, collected a large dataset of five million weakly labeled images. Gender recognition through face segmentation is already explored in another work [67,68]. However, the work proposed in [67,68] has been validated on very limited data and through traditional machine learning methods.

### 2.5. Multi Tasks Framework

A framework addressing gender and age was prosed by Toews and Arbel [69]. The proposed model is a view-point invariant appearance model that is robust to rotations at the local scale level. Another algorithm proposed by Yu et al. [70] was based on gait and linear discriminant algorithms. A benchmark for both age and gender was proposed in [71]. Khan et al. [72] suggested another algorithm, also called semantic pyramid gender and action recognition method, which addressed both gender and action recognition. Chen et al. [43] proposed a multi modeling mechanism, which combined both the text and image information. Higher accuracy was reported as compared to SOA with the proposed model. Another generic algorithm proposed in [46] estimated gender, race, and age in a single framework.

The performance of different visual recognition tasks was much improved with recently introduced deep learning architectures. The three demographic attributes (race, age, and gender) were also explored in a single model through these deep learning architectures. For example, a hybrid approach for age and gender was introduced in [73]. DCNNs were used for features extraction and for classification extreme machine learning (EML) strategy was adapted. The proposed method was named CNNs-ELMs due to the joint venture of DCNNs and EML. The CNNs-ELMs was evaluated on two challenging databases MORPH-II [59] and Adience [71].

All the methods mentioned above made lots of progress towards mature face image analysis systems. However, these methods were designed either for non-automated estimation algorithms or worked well in constrained and controlled imaging conditions. Both appearance and geometric based methods were facing some serious problems, we approached face image analysis through a different idea. Our face image analysis and attributes classification idea is novel; in a sense, we approach the face analysis task through a prior face segmentation method. Initially, we segment a face image into seven parts, including mouth, hair, back, skin, nose, eyes, and eyebrow. We used a probabilistic classification strategy and modeled a DCNNs based framework for each demographic task, i.e., race, age, and gender recognition. We test our framework on SOA databases, obtaining superior results as compared to previous results.

## 3. Used Datasets

In this Section of the paper, we discuss different face image databases we used to evaluate our framework.

### 3.1. Face Parsing

**Figure 1:**
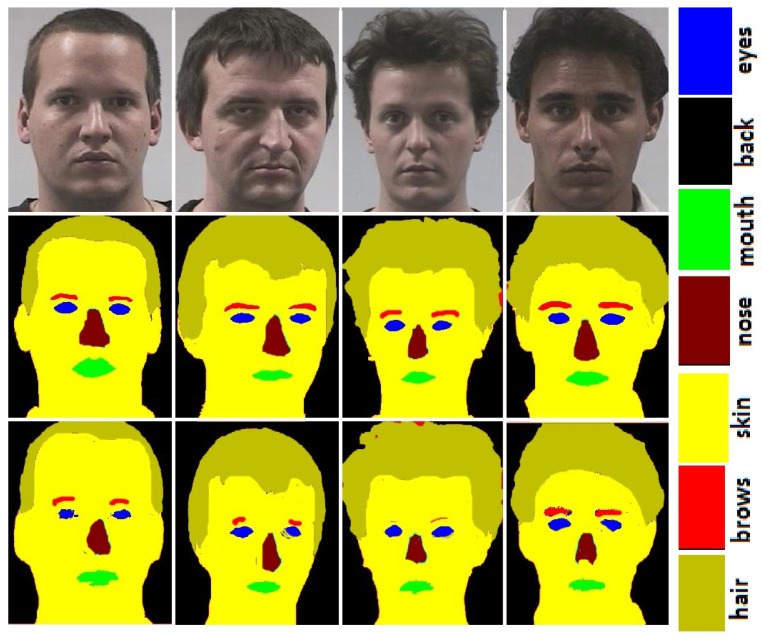
Face parsing results for FASSEG [29] frontal images. First row show: original images, second row: ground truth, and third row: face parsing results.

**Figure 2:**
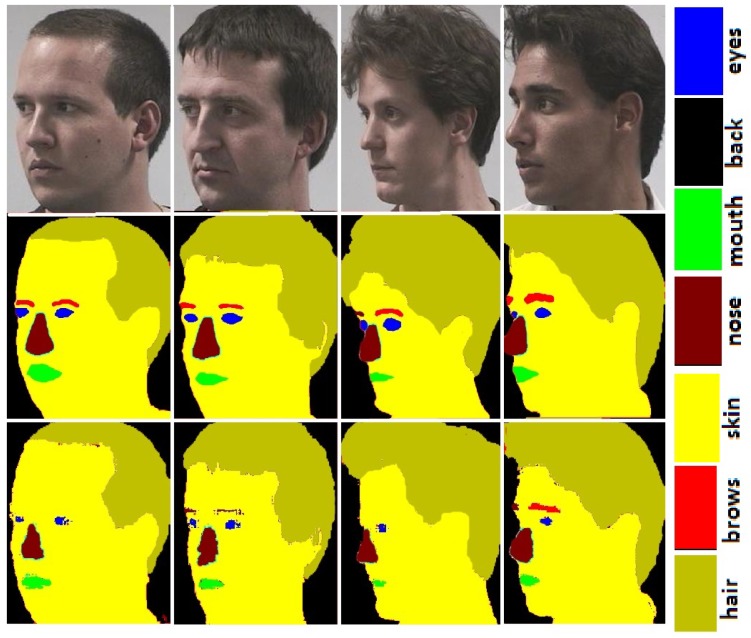
Face segmentation results for profile images for FASSEG [29] database. Order of the images, row 1 shows: original images, row 2: ground truth, and row 3: face parsing results.

To the best of our knowledge, three authentic databases are publically available for different face parts labeling. Details of these datasets are as follow;

- **LFW-PL**: We evaluate our face parsing part with LFW-PL [27]. Some recent methods [26,74] already use the LFW-PL [27] for face parsing. We use a subset of training and testing images. For fair and more exact comparisons, we conduct experiments on the same set of images as in [75]. The LFW-PL contains 2927 images with size 250×250, which are all collected in the wild conditions. The ground truth data are created manually through commercial editing software. All face images are labeled to three classes, including back, skin and hair.
- **HELEN:** The HELEN [28] database contains class labels for 11 categories. This database contains 2330 images, each with size 400×400. These images are also manually labeled. The database is divided into a training set (2000) and a validation set (330). We keep the experimental setup as in [75]. Although the HELEN database is a comparatively large database having 11 dense classes, the ground truth labeling is not very precise. Especially the hair class is mostly mislabeled with skin in most of the cases.
- **FASSEG:** The FASSEG [29] consists of both frontal and profile face images. Frontal01, frontal02, and frontal03 contain frontal images of 220 faces along with ground truth data. The subset multipose01 contains profile face images of more than 200 faces. The FASSEG images are taken from other publically available datasets, and ground truth data is created through manual editing tool. The images contain both high and low-resolution data. The illumination conditions and facial expressions are also changing in some cases. The dataset is very precise as ground truth data is created with extreme care. Figure 1 and Figure 2 show some images from the FASSEG [29] database. Original images are shown in row 1, ground truth in row 2 and the segmentation results in row 3.

### 3.2. Race, Age, and Gender

**Figure 3:**
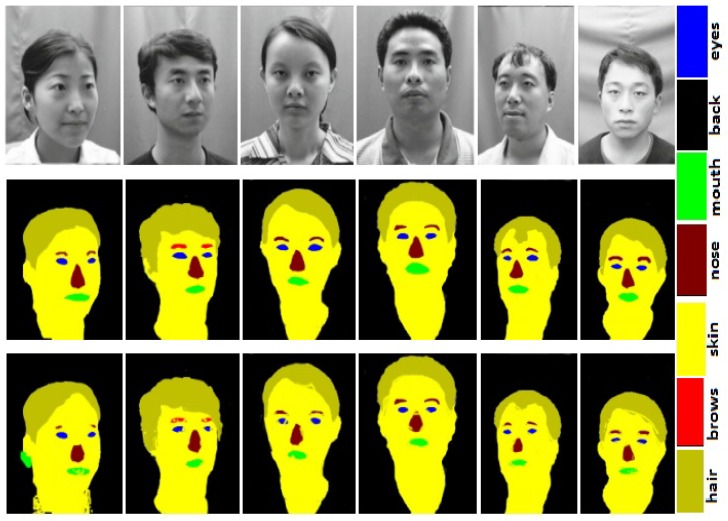
Face images from CAS-PEAL [32] dataset in row 1, ground truth in row 2, and face segmentation results in row 3.

**Figure 4:**
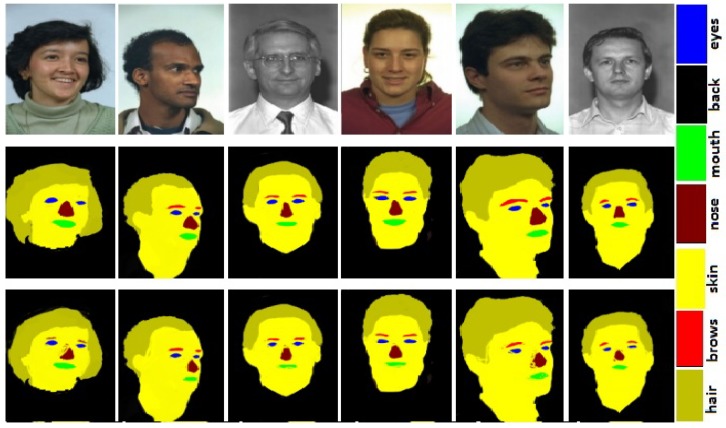
Original face images from FERET [31] database in row 1, ground truth in row 2, and face parsing results in row 3.

- **CAS-PEAL** The CAS-PEAL [32] is a face database used for various tasks such as head pose estimation, gender recognition, race classification, etc. It is a larger dataset with 99,594 face images. The CAS-PEAL [32] is collected with a large number of face images having 1040 subjects. The dataset is sufficiently large, but the complexity level of the images is not higher, making the dataset a bit simple. We used CAS-PEAL [32] for race classification in the proposed work. Figure 3, row 1 shows some images from the CAS-PEAL [32] database. The ground truth images manually labeled to build DCNNs model are shown in row 2, whereas the segmentation results with proposed DCNNs model are shown in row 3.
- **FERET:** This is an old dataset which is used for various face analysis tasks such as face recognition, head pose estimation, gender recognition, etc. The FERET [31] dataset is collected in very constrained lab conditions, and gender information is also provided for each participant. It is a medium-sized dataset with 14,126 face images. However a sufficient number of participants are included in the database collection with 1199 subjects. We use the colored version of the FERET [31]. The participants include variations in facial expressions, changing in lighting conditions, which make the database a bit challenging. we evaluate our race and gender recognition part with FERET [31] database. Figure 4, row 1 shows some face images from the FERET [31] database. The ground truth images manually annotated are shown in row 2, whereas the segmentation results in row 3.
- **LFW:** The LFW [76] database consists of 13,233 face image collected from 5749 participants. The dataset is collected in very unconstrained environmental conditions. All the face images are collected from the internet, with very poor resolution. The LFW [76] is a very unbalanced database, as the number of female participants are 2977, whereas male candidates are 102,566. We use this database for evaluating our gender recognition part.
- **Adience:** The Adience [71] is a new database that was released in 2018. We evaluate our age and gender classification part with Adience [71]. The database is collected in the wild and real-world conditions. The images are collected through smart phones Much complexities are added to the images to make the database rather challenging; such as pose variation, changing lighting conditions, noise, etc. are present in the images. It is a comparatively larger dataset with more than 25,580 face images. Sufficient number of candidates are included (2284) in the dataset collection. Information about the exact age of each participant is not provided, instead each participant is assigned to eight age groups, i.e., [0,2], [4,6], [8,13], [15,20], [25,32], [38,43], [48,53], [60,+]. The database is freely available for downloading from the Open University of Israel.

## 4. Proposed Face Parsing Framework (MCFP-DCNNs)

In this Section of the paper, we present the DCNNs we used to build our face parsing model. We make this model for each demographic task, i.e., race, age, and gender.

Face parts are not localized in face images with some datasets. We apply a face detection algorithm in the start if needed. Many excellent face detection algorithms are reported in the literature; we use a deep learning-based face detector reported in [77]. After face detection, we re-scaled all face images to a fixed size 227×227. Details of the proposed DCNNs architecture is in the following paragraphs;

### Architecture

The performance of the DCNNs based model depends on several parameters; for example, the size of the kernels used, the convolutional layer numbers, and filters in every layer. In our DCNNs model, we used three convolutional (C1–C3) layers, each followed by a max-pooling layer (P1–P3). The size of the kernel in the convolutional layer was set as 5×5. The down sampling stride for both convolutional and max-pooling layer was fixed at two. We kept the kernel size 3×3 in the max-pooling layer. Table 1 shows details about each convolution layer, kernel size, stride, and feature maps. Various parameters setting of the proposed CNNs is presented in Table 2.

For activation function we used rectified linear unit (ReLu). After each convolutional layer we embedded pooling layer. For pooling layer we used max-pooling.

A complete DCNNs model has three main parts, i.e., convolutional layers, pooling layer, and fully connected layers. We represented the kernels as N×M×C where N and M represent the height and width of the filter and C represents the channel. The pooling layers filters are represented by P×Q, where *P* represents height and Q width of the filter. The fully connected layer is the final layer which performs the task of classification. For better optimization of the DCNNs and more exploring deep learning architecture, readers are recommended to read Goodfellow et al.’s book [78].

## 5. Proposed RAG-MCFP-DCNNs

Initially, we develop a segmentation model MCFP-DCNNs for each demographic task, i.e., race, age, and gender. The MCFP-DCNNs assigns a semantic class label to each pixel of a face image. We use a probabilistic classification strategy and generated probability maps (PMAPs) for each face class. The PMAPs are computed by converting the probability of each pixel to a gray-scale image. In PMAPs, higher intensity represents a higher value of probability for the most likely class on their respective position and vice versa.

**Figure 5:**
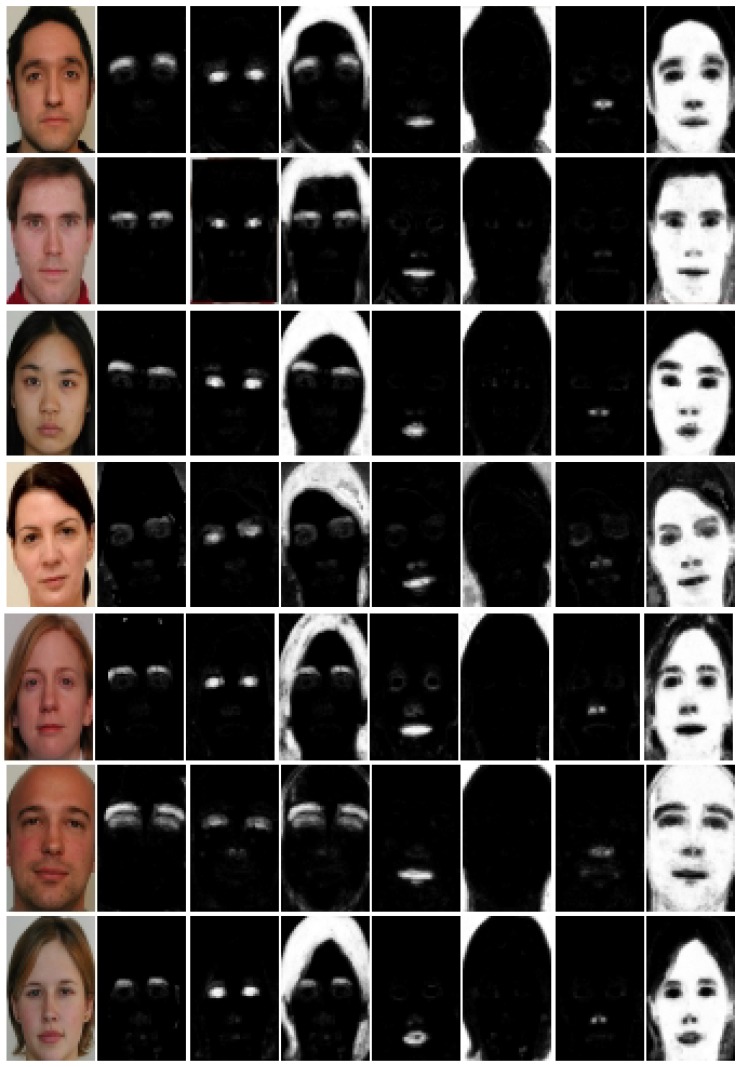
Example face images from FASSEG [29] database, probability maps in the order such that: column 1 shows: original images, 2: eyebrow, 3: eyes, 4: hair, 5: mouth, 6: back, 7: nose, and 8: skin class.

Figure 5 shows some images of FASSEG [29] database and their respective probability maps for seven classes. The better segmentation for a specific class, the higher will be the predicted probability value and vice versa. As a result, a brighter PMAP on the respective position will be obtained. From Figure 5, it is clear that some good results are produced by the segmentation model for skin, back, and hair. These regions can be easily differentiated from the others in a respective PMAP image, as can be seen from column 4, 6, and 8 (PMAPs for hair, back, and skin) in Figure 5. The segmentation results also confirm this fact because much better results are obtained for these classes as compared to minor classes. On the other hand, PMAPs for minor classes can not be differentiated from the remaining parts in a respective PMAPs, leading to a confusing situation. PMAPs for minor classes are shown in column 2, 3, 5, and 7 (brows, eyes, mouth, and nose).

We investigated thoroughly which PMAPs are more helpful in age, race, and gender classification. The PMAPs which are helpful for the respective task are then used for feature extraction.

We presents summary of the proposed RAG-MCFP-DCNNs in Algorithm 1. Initially a segmentation model is developed through CNNs. For the classification of race, age, and gender we use PMAPs created during segmentation. We use these probability maps as features descriptors. We extracted features from these PMAPs through deep convolutional neural networks. After extracting features from PMAPs, feature vectors for the corresponding classes are concatenated to a single unique feature vector which is given to Soft-Max classifier. We use 10-fold cross validation experiments in our work. We represent PMAPs generated for each semantic class as ***PMAP*****${}_{nose}$**, ***PMAP*****${}_{eyebrow}$**, ***PMAP*****${}_{back}$**, ***PMAP*****${}_{mouth}$**, ***PMAP*****${}_{eyes}$**, ***PMAP*****${}_{skin}$**, and ***PMAP*****${}_{hair}$**.
**Algorithm 1** proposed RAG-MCFP-DCNNs algorithm**Input:*****M*****${}_{train}$** = {(*I${}_{i}$*,*T${}_{i}$)*}${}_{i=1}^{j}$, ***M*****${}_{test}$** where the DCNNs model is trained through training data represented as ***M*****${}_{train}$** and tested through ***M*****${}_{test}$**. The input training image is represented as *I* and the ground truth data is *T(i,j)*∈ {1,2,3,4,5,6,7}. **a: Face parsing part:** Step a.1: Training a face parsing model DCNNs through training images and class labels. Step a.2: Using the probabilistic classification strategy and producing PMAPs for each semantic class, represented as: *PMAP*${}_{skin}$, *PMAP*${}_{mouth}$, *PMAP*${}_{eyes}$, *PMAP*${}_{nose}$, *PMAP*${}_{hair}$, *PMAP*${}_{back}$, and *PMAP*${}_{eyebrow}$ **b. race, age and gender classification part:** Training a second DCNNs for each demographic class (race, age, and gender) by extracting infomration from PMAPs of the corresponding classes such that; **if** race classification:  f=*PMAP*${}_{skin}$ + *PMAP*${}_{mouth}$ + *PMAP*${}_{eyes}$ + *PMAP*${}_{nose}$ + *PMAP*${}_{hair}$ + *PMAP*${}_{brows}$ **Else if** age classification:  f=*PMAP*${}_{skin}$ + *PMAP*${}_{mouth}$ + *PMAP*${}_{eyes}$ + *PMAP*${}_{nose}$ + *PMAP*${}_{brows}$ **Else if** gender recognition:  f=*PMAP*${}_{skin}$ + *PMAP*${}_{eyes}$ + *PMAP*${}_{brows}$ + *PMAP*${}_{nose}$ + *PMAP*${}_{mouth}$ where f is the feature vector. **Output:** estimated race, age and gender.


For each face analysis task (race, age, and gender), we train a second DCNNs using the corresponding PMAPs as features descriptors. The DCNNs extract features from the corresponding classes which are used to train and test race, age, and gender classification module.

### 5.1. Race Classification

We classify face images into two races, i.e., Asian and Non-Asian. For race classification, we used two datasets, namely CAS-PEAL [32] and colored version of FERET [31]. The CAS-PEAL [32] is a Chinese database containing images collected in different poses. We named images of CAS-PEAL [32] as Asian class. The colored version of FERET [31] contains 12,332 face images. All images in FERET [31] are Non-Asian; hence, we named these images as Non-Asian class. Sample face images from both CAS-PEAL [32] and FERET [31] are shown in Figure 3 and Figure 4.

We manually labeled 200 face images from each race class of each database. We used the manually labeled images to build an MCFP-DCNNs model, as discussed in Section 4. For all face images of each database, we generated PMAPs. When a test face image was provided as input to the RAG-MCFP-DCNNs, the model predicted PMAPs from the segmentation part for all seven face classes.

**Figure 6:**
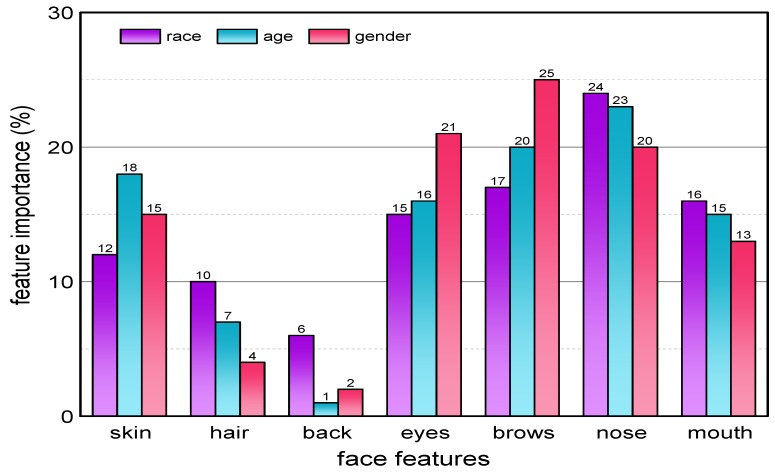
Feature importances of ‘skin’, ‘hair’, ‘back’, ‘mouth’, ’eyes’, ’brows’, and ‘nose’ for three facial attributes (race, age, and gender) classification.

To know which face parts help in race classification, we conducted a set of qualitative and quantitative experiments. A graph in Figure 6 shows which face part contributes towards race classification. From the Figure 6 it is clear that six face classes contribute towards age classification. We utilized PMAPs for eyes, nose, mouth, skin, eyebrows, and hair. We extracted features from PMAPs of the above mentioned six classes through DCNNs. For classification we used Soft-Max classifier as in the first case. We kept 10-fold cross validation experimental setup for race classification. Images that were used to build the MCFP-DCNNs model were excluded from the testing phase.

### 5.2. Age Classification

In our age classification module, each face image is given a specific age category within certain categories. We manually labeled 20 images from each age group. We used the manually labeled images to build an MCFP-DCNNs model for age classification.

We investigated during experiments that each face part has certain contribution towards age classification. Figure 6 shows how different features contributes to the age classification. From Figure 6, it is clear that skin, nose, eyes, mouth, and eyebrows contributes significantly towards age classification. We also notice that using all class information makes the algorithm computationally quite expensive. Therefore, we used a subset of all the seven classes for age classification.

All the testing images were passed to the MCFP-DCNNs to get a probability value for each class. We generated PMAPs for each age category image and each class. We extracted features from the PMAPs through DCNNs. For all face images of each database, we generated PMAPs. When a test face image is provided as input to the MCFP-DCNNs, the model predicts PMAPs from the segmentation part for all seven face classes.

We used Soft-Max for classification, as previously. We kept 10-fold cross validation experimental setup during our experiments. We excluded face images that were previously used to build the MCFP-DCNNs model for age classification.

### 5.3. Gender Recognition

We manually labeled 50 images from each gender for gender recognition. We built a DCNNs based model for gender classification. We performed intensive experiments to know which face parts help in gender classification. Human face anatomy also helps in gender classification. These fundamental differences also help us to develop a gender classification module; the information is summarized as follows:

- Face anatomy reports that the male forehead is more significant than the female forehead. In most of the cases, male hairline lags behind as compared to female. Is the case of baldness (males only) hairline is missing entirely. All this results in a more massive forehead in males compared to females. We assigned to all forehead a skin label. Hence our MCFP-DCNNs model creates a probability map for skin, which is on the larger brighter area in males compared to females.
- Visually, female eyelashes are curly and comparatively larger. These eyelashes are misclassified with hair and in some cases with eyebrows. Although, labeling accuracy is reduced for segmentation part with this misclassification, however, this misclassification helps in gender classification. The MCFP-DCNNs model generated a brighter PMAPS for males as compared to females.
- Qualitative results reveal that the male nose is larger than the female nose. The male body is larger, which needs a sufficient supply of air towards the lungs. This results in larger nostrils and a giant nose for males compared to females. We also encode this information in the form of PMAPs through MCFP-DCNNs.
- Literature reports very complex geometry for hair. For humans, it is easy to identify the region between hair and face parts, but for the computer, it is not an easy task. Our MCFP-DCNNs model reports excellent labeling accuracy for hair class. From segmented images it can be seen how efficiently hairline is detected by MCFP-DCNNs. We also encoded this information in PMAPs for hair class and used it in gender classification.
- Eyebrows is another class that helps immensely in gender classification. It is generally noticed that female eyebrows are thinner, well managed, and curly at the ends. On the other hand, male eyebrows are thicker, mismanaged. We obtained better labeling accuracy for eyebrow from our face parsing model.
- Mouth is another class that also helps in gender recognition. Female lips are visible and very clear; in the male in some cases (in images), the upper lip is even missing. We encoded this information as well and used it in our modeling process.

Due to the reasons mentioned above, we use PMAPs of five classes including skin, nose, eyes, brows, and mouth to build the second stage of DCNNs model. We perform 10-fold cross-validation experiments to evaluate our model more precisely. We excluded all those images from the testing phase, which were previously used to build MCFP-DCNNs.

## 6. Results and Discussion

### 6.1. Experimental Setup

**Hardware Platform:** For experiments we used intel i7 CPU. RAM of the system was 16 G while graphical processing unit was NVIDIA 840 M. We used TensorFlow and Keras for experiments. We trained the model for 30 Epochs and the batch size was 125.

**Figure 7:**
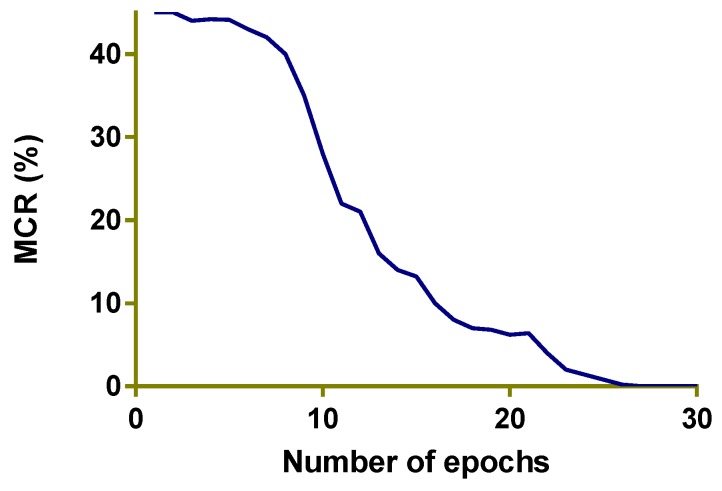
The mis-classification rate (%) for training data (for HELEN [28] database only).

From Figure 7 it is clear that the mis classification rate reduces as the number of Epochs are increased. At 25 Epochs the miss classification rate for training data almost reaches to 0. This graph is for training data of the face parsing part for HELEN [28] database only.

### 6.2. Face Parsing Results

Previously, FASSEG [29] was evaluated with pixel labeling accuracy (PLA). The PLA compares the estimated segmentation with manually annotated labels. For fair comparison with SOA We also report the face parsing results in the form of confusion matrices.

F-measure is a common metric used in the literature for evaluating a face parsing framework. We used F-measure for our evaluation of our work fot two dataset LFW-PL [27] and HELEN [28]. We used the same setting as in [48,75,79]. The total face images in HELEN are 2330. We used 2000 for training, 230 for the validation, and remaning 100 images for the testing phase.

The LFW-PL [27] is a database with 2972 face images. All these images were manually annotated with three labels: hair, back, and skin. As in [21,27], we also used 1500 images for training, 500 for validation, and 927 for the testing phase. However, we re-annotate the remaining four parts, namely, nose, eyebrow, mouth, and eyes. We reprocess the already annotated parts if needed, as the manual annotation is not precise for LFW-PL [27]. For more accurate comparison, we kept the testing part images unchanged except adding additional four parts labels for our own experiments.

We summarize the key points of the face segmentation results as following:

**FASSEG:** Some face Images from FASSEG are shown in Figure 1 and Figure 2. Images in Figure 1 are showing some segmentation results; we categorize these as good results. Original face images are placed in the first row, ground truth in the second row, and segmented images in row three. It is also clear from these images that better segmentation is produced for frontal images as compared to profile images, as expected. It can also be observe from these images that better segmentation results are produced for larger classes (skin, hair, and back) while comparatively poor segmentation results are noticed for smaller classes (eyes, nose, mouth, and eyebrows).

There is very limited research work on FASSEG [29] database. Results reported for FASSEG till date are shown in Table 3. From the Table 3 it is clear that we have much better results as compared to previous results. The results reported in [80] do not consider an eyebrows class; we also added eyebrow parts in our current research work.

**HELEN:** Previously, background class was not considered in face parsing. We believe sometime background class also helps in some real-world application scenarios. We included background class also and reported results in our paper. Results reported with our proposed face parsing model and its comparison with SOA are shown in Table 4. From Table 4, it is clear that most of the previous work does not consider hair class in segmentation due to its complex geometry. We included hair class as well in our face parsing model.

It is clear from Table 4 that we have better results as compared to previous results. However, the performance of the proposed MCFP-DCNNs is poor with minor classes (eyes, brow, and mouth). We obtained better results on three major classes (skin, hair, and back) and one minor class (nose). However, the overall results for the face parsing is improved as the contribution of the major classes in the face is more as compared to minor classes.

**LFW-PL:** Results for the LFW-PL [27] database is reported in Table 5. From the Table 5, it is clear that we have improvements in results for two major classes, hair and background. However, previous results surpassed us in one case [23], as can be seen. The overall performance is also comparatively poor, as compared to previous results [23]. The possible reason: we included four smaller classes which are comparatively difficult and lower PLA as well. The lower PLA values for smaller classes decreased the overall performance of the framework as well. We also noted the poor performance of the prosed MCFP-DCNNs for background class for LFW-PL [27] database. This confirms the fact that the proposed algorithm is not working good with complicated background scenarios, as LFW-LP [27] images have complex background.

We also noticed that segmentation is highly depended on the quality of face images. For example, we noted poor segmentation results for LFW-PL [27] and much better results on FASSEG [29] and HELEN [28].

#### Race, Age, and Gender Classification Experimental Setup

In this Subsection, the experimental setup for training and testing data of race, age, and gender classification is presented.

**Race classification:** For race classification, we used two datasets, including FERET [31] and CAS-PEAL [32]. We selected 100 images randomly from each dataset. The 200 chosen images were excluded from the testing phase of race classification.

**Gender classification:** We used three datasets for gender recognition, including LFW [76], Adience [71], and FERET [31]. We selected 50 images each from these datasets, constituting a total of 150 images. As in race classification, the training phase images were excluded from the testing phase.

**Race Classification:** We used the Adience [71] dataset for age classification. The Adience dataset contains eight different age categories. We manually labeled 20 images from each group. In this way, the total number of training images we selected were 160. In all the three cases above, the selection of the images for the training phase was random. The training phase images were not included in the testing phase. Moreover, to validate the model and results more precisely, we conducted 10-fold cross validation experiments for all the three cases (race, age, and gender classification).

### 6.3. Race Classification

To know how much each facial part contributes to specific demographic task, we exploited the feature importance measure, which is returned by a Random Forest implementation as in [85]. Figure 6 shows the feature importance of all the facial parts for all three tasks. From Figure 6, it is clear that the nose has a maximum and background minimum contribution towards race classification.

We report race classification results with classification accuracy. For race classification, we used two datasets, namely CAS-PEAL [32] and FERET [31]. The first database represents Asian and the later Non-Asian class. We manually annotated 100 images from each of these databases for training a DCNNs based model.

The MCFP-DCNNs built for the race was used to create PMAPs for each image in the testing phase. We created PMAPs for all images of both classes. We built another DCNNs using the PMAPs as descriptors and extracting features from the corresponding PMAPs. We performed 10-fold cross-validation experiments in our work. We excluded 200 images that were previously used to train MCFP-DCNNs.

For race classification, we investigated the possible combination of facial features. We noticed during these experiments the contribution of each face part towards race classification. We utilized six face features, excluding background to train a second stage of DCNNs.

We reported results and comparison with SOA in Table 6. From Table 6, it is clear that we have perfect results for Asians and better results as compared to previous results for the Non-Asian class.

From Table 6, it is clear that we used only two classes (Asian and Non-Asian) for experimentation. Although we evaluated our work on two large databases, but the number of races to be classify were limited.

The computational cost is another factor that we did not consider in our work. One main limitation of the deep learning architectures is a substantial computational cost, which we also faced in our work. Our approach may lag as compared to previous methods if compared computationally, as we built two DCNNs models for complete face analysis.

### 6.4. Age Classification

We reported our age classification results with classification rate, as in race classification. We used Adience [71] for age classification. This database has eight different age categories. We labeled 20 face images from each category. We built our age classification model with 160 manually labeled images.

The MCFP-DCNNs model was used to create PMAPs for each testing image. After creating PMAPs for all images and all eight classes, we performed 10-fold cross-validation experiments. We excluded all 160 images from the testing phase, which were previously used to build an MCFP-DCNNs model.

We investigated all possible combinations of facial features for age classification. Figure 6 shows which face part has major contribution toward age classification. Again nose has most contribution and back least contribution towards age classification. We used all six face classes, excluding the background class to built a DCNNs model.

We show our reported results and its comparison with SOA in Table 7. It can be seen from Table 7 that we have much better results as compared to SOA on age classification for Adienece [71] database.

We manually labeled ground truth data using an image editing software. We did not use any automatic manually labeling tool. This ground truth labeling has two significant drawbacks. First, such sort of labeling highly depends on the subjective perception of a subject who is involved in all labeling process. Accurate label providing in such cases is tough, specifically differentiating the boundary region between two or more regions is highly challenging. For example, it is very difficult to distinguish the skin region from the nose and vice versa. Second, this ground truth labeling is a very time consuming process. One main drawback of our proposed method is; this research work is limited to age classification only, which is due to tedious labeling process. We do not consider age estimation, because, in that case, a large number of images needed to be labeled. Moreover, the computational cost of the framework will also be sufficiently large.

### 6.5. Gender Recognition

As in the other two cases, we try all possible combinations of facial features for gender classification. After experimentation, we conclude to use five parts, i.e., nose, mouth, eyebrows, eyes, and skin. Figure 6 shows contributions of each face part in gender classification. We created manually labeled images from each male and female gender. We randomly took 30 images from each gender and each database to train an MCFP-DCNNs model. The total training images were 180. As in the case of age classification, we excluded 180 images that were previously used to build an MCFP-DCNNs model.

We perform gender classification with three datasets, including Adience [71], LFW [76], and FERET [31]. In Table 8, we show classification accuracy for all the three datasets. Table 8 also compares our reported results with SOA. For gender classification, we perform 10-fold cross-validation experiments, as in the previous two cases. We obtained better results as compared to previous results, as can be seen from Table 8.

As a whole, we noticed the performance of the proposed RAG-MCFP-DCNNs very interesting. We introduced an idea of human face image analysis, which is using different face parts information provided by a segmentation model. We reached an important observation stating: *“face parts parsing and different visual recognition tasks are closely related, the better segmentation, better results for the three tasks will be observed".*

## 7. Conclusions

We proposed an end-to-end face parsing method which tries to address three face image analysis tasks, including race, age, and gender classification. We trained the MCFS-DCNNs model through a DCNNs model by extracting information from various face parts. The MCFS-DCNNs classified every pixel to one of the seven categories (hair, eyebrows, eyes, skin, nose, back, and mouth). We used probabilistic classification method to generate PMAPS for seven face classes. We built another DCNNs model by extracting features from the corresponding PMAPs for each of the three demographic tasks (race, age, and gender). We performed a series of experiments to investigate which face parts help in the race, age, and gender classification. We validate our experiments on seven face databases, obtaining much better results as compared to SOA.

We argue that sufficient information is provided by the face parsing model for different visual recognition tasks. We provide a route towards other complicated face image analysis problems. For example, we intend to add complicated facial expressions, head pose estimation, and many other applications to the framework. We are also planning to optimize the segmentation part to improve the performance of the face parsing part of the framework. 

## Figures and Tables

**Table 1:** Information about each convolutional neural networks (CNNs) layer.

Layer	kernel Size	Stride	Feature Maps	Output Size
Input image	–	–	–	227×227
C-1	5×5	2	96	112×112
P-1	3×3	2	96	56×56
C-2	5×5	2	256	27×27
P-2	3×3	2	256	12×12
C-3	5×5	2	512	5×5
P-3	3×3	2	512	2×2

**Table 2:** Parameters setting for CNNs training.

Parameters	Vales
Epochs	30
Batch size	125
Momentum	0.9
Base learning rate	$10^{-4}$

**Table 3:** Face parsing: comparison of the MCFP-DCNN with SOA on FASSEG (frontal) DB. The reported results are based on pixel labeling accuracy.

Method	Eyes	Brows	Mouth	Nose	Skin	Hair	Back	Overall
Khan et al. [80]	60.75	–	84.2	61.25	94.66	95.81	91.50	–
MCFP-DCNNs	84.30	86.25	89.30	87.8	96.58	98.2	94.54	95.12

**Table 4:** Face parsing: comparison of the MCFP-DCNN with SOA on HELEN DB. The reported results are for F1 measure.

Method	Eyes	Brows	Mouth	Nose	Skin	Hair	Back	Overall
Smith et al. [24]	78.5	92.2	85.7	92.2	88.2	–	–	80.4
Zhou et al. [81]	87.4	81.3	92.6	95.0	–	–	–	87.3
Liu et al. [75]	76.8	71.3	84.1	90.9	91.0	–	–	84.7
Liu et al. [22]	86.8	77.0	89.1	93.0	92.1	–	–	88.6
Wei et al. [48]	84.7	78.6	89.1	93.0	91.5	–	–	90.2
Jonathan et al. [23]	89.7	85.9	95.2	95.6	95.3	88.7	–	93.1
MCFP-DCNN	78.6	83.2	88.5	97.2	96.2	98.4	86.2	95.2

**Table 5:** Face parsing: comparison of the MCFP-DCNN with SOA on LFW-PL DB. The reported results are for F1 measure.

Method	Eyes	Brows	Mouth	Nose	Skin	Hair	Back	Overall
Liu et al. [75]	–	–	–	–	93.93	80.70	97.10	95.12
Long et al. [82]	–	–	–	–	92.91	82.69	96.32	94.13
Chen et al. [83]	–	–	–	–	92.54	80.14	95.65	93.44
Chen et al. [84]	–	–	–	–	91.17	78.85	94.95	92.49
Zhou et al. [81]	–	–	–	–	94.10	85.16	96.46	95.28
Liu et al. [22]	–	–	–	–	97.55	83.43	94.37	95.46
Jonathan et al. [23]	–	–	–	–	98.77	88.31	98.26	96.71
MCFP-DCNN	78.2	68.3	72.5	85.7	96.8	94.2	97.2	93.25

**Table 6:** Comparison between proposed method and related works on race classification.

Database	Method	Asian (Accuracy%)	Non-Asian (Accuracy%)
RAG-MCFP-DCNNs	–	100	96.4
Manesh et al. [38]	FERET [31] and CAS-PEAL [32]	98	96
Muhammed et al. [86]	FERET [31]	99.4	–
Chen and Ross [43]	CAS-PEAL [32]	98.7	–
Anwar and Naeem [42]	FERET	98.28	–

**Table 7:** Comparative experiments on age classification using Adience database.

Database	Method	Classification Aaccuracy (%)
Adience	RAG-MCFP-DCNNs	69.4
	Dehghan et al. [87]	61.3
	Hou et al. [88]	61.1
	Hassner et al. [89]	50.7
	Hernandez et al. [90]	51.6
	CNN-ELM [73]	52.3

**Table 8:** Comparative experiments on gender recognition using Adience, LFW and FERET data-sets.

Database	Method	Classification Accuracy (%)
Adience	RAG-MCFP-DCNNs	93.6
	Levi et al. [91]	86.8
	Lapuschkin et al. [92]	85.9
	CNNs-EML [73]	**77.8**
	Hassner et al. [89]	79.3
LFW	Van et al. [93]	94.4
	RAG-MCFP-DCNNs	94.1
	HyperFace [94]	94.0
	LNets+ANet [95]	94.0
	Moeini et al. [96]	93.6
	PANDA-1 [47]	92.0
	ANet [56]	91.0
	Rai and Khanna [97]	89.1
FERET	RAG-MCFP-DCNNs	100
	Moeini et al. [96]	99.5
	Tapia and Perez [98]	99.1
	Rai and Khanna [97]	98.4
	Afifi and Abdelrahman [99]	99.4
	A priori-driven PCA [100]	84.0
